# Development and validation of a questionnaire evaluating patient anxiety during Magnetic Resonance Imaging: the *Magnetic Resonance Imaging-Anxiety Questionnaire (MRI-AQ)*

**DOI:** 10.1186/1532-429X-18-S1-P312

**Published:** 2016-01-27

**Authors:** Britt-Marie Ahlander, Kristofer Arestedt, Eva Maret, Jan E Engvall, Elisabeth Ericsson

**Affiliations:** 1 grid.5640.70000000121629922Department of Medical and Health Sciences, Linkoping University, Linkoping, Sweden; 2 grid.5640.70000000121629922Center of Image Science and Visualization, Linkoping University, Linkoping, Sweden; 3 grid.413253.2Department of Radiology, Ryhov County Hospital, Jonkoping, Sweden; 4 grid.24381.3c0000000092415705Department of Clinical Physiology, Karolinska University Hospital, Stockholm, Sweden; 5 grid.8148.50000000121743522Center for Collaborative Palliative Care, Linnaeus University, Kalmar, Sweden; 6 grid.15895.300000000107388966Faculty of Medicine and School of Health and Medical Sciences, Orebro University, Orebro, Sweden

## Background

MR examinations of the heart are sometimes negatively affected by anxiety that could have been detected by the pre-scan administration of a suitable questionnaire and psychological support given. To better understand patient experience during the examination and to evaluate psychological intervention, use of general anxiety scales is unsuitable and an MR-specific questionnaire was developed.

## Methods

A new questionnaire, MRI-AQ, was designed from patient expressions of anxiety in MRI-scanners. The patient sample was recruited between October 2012 and October 2014. Factor structure was evaluated with exploratory factor analysis and internal consistency with Cronbach's alpha. Criterion-related validity, known-group validity and test-retest was evaluated. The new instrument was compared with the Spielberg State Anxiety Index (STAI), the Hospital Anxiety and Depression Scale (HAD), and with nine statements from the Fear Survey Schedule developed by Lukins et al.

## Results

In total, 247 participants (54.7 ± 14.3 years), referred for MRI examinations of either the spine or the heart, accepted to participate in the study. The development and validation of MRI-AQ resulted in 15 items which could be used as an overall global score or as two sub scale scores. Cronbach's alpha was found to be high (α = 0.90). MRI-AQ correlated higher with instruments measuring anxiety than with depression scales. Known-group validity demonstrated a higher level of anxiety for patients undergoing MRI scan of the heart than for those examining the spine (p < 0.01). Test-retest reliability demonstrated acceptable level for the scale (ICC = 0.90; CCC = 0.90).

## Conclusions

MRI-AQ bridges a gap among existing questionnaires, making it a simple and useful tool for measuring patient anxiety during MRI examinations.

**Table 1:** MRI-AQ compared with general anxiety scales

Scale/factors	MRI-FSS	STAI-S	HAD-A	HAD-D	Patient Experience	Patient Worry
MRI-AQ, total scale	0.40***	0.50***	0.26***	0.16*	0.66**	0.80***
MRI-AQ reduced scale	0.41***	0.48***	0.28***	0.16 *	0.66**	0.82**
Anxiety symptoms	0.43***	0.44***	0.27***	0.14	0.69**	0.84**
Relaxation symptoms	0.41***	0.40***	0.28***	0.14	0.53***	0.71**
Reliance on staff	-0.05	0.12	-0.08	0.02	0.00	-0.08

Total score MRI-AQ= Magnetic Resonance Imaging- Anxiety Questionnaire, MRI-FSS= Magnetic Resonance Imaging Fear Survey Schedule, STAI-S = Spielberg State Anxiety Index- State, HAD-A= Hospital Anxiety and Depression Scale -Anxiety, HAD-D= Hospital Anxiety and Depression Scale - Depression. Patient experience and patient worry=10 point scale *P < 0.05; **P < 0.01; ***P < 0.001. Person's correlation (r).

